# Effects of dabigatran and rivaroxaban on stroke severity according to the results of routine coagulation tests

**DOI:** 10.1371/journal.pone.0240483

**Published:** 2020-10-12

**Authors:** Han-Jin Cho, Yoon Jung Kang, Sang Min Sung, Sung-Ho Ahn, Yo Han Jung, Kyung-Yul Lee, Jung Hwa Seo, Sang Won Han, Joong Hyun Park, Hye-Yeon Choi, Jee-Hyun Kwon, Wook-Joo Kim, Hyung Jong Park, Jin Kyo Choi, Hyo Suk Nam, Ji Hoe Heo, Young Dae Kim

**Affiliations:** 1 Department of Neurology, Pusan National University Hospital, Pusan National University School of Medicine and Biomedical Research Institute, Busan, Korea; 2 Department of Neurology, Pusan National University Yangsan Hospital, Pusan National University School of Medicine, Yangsan, Korea; 3 Department of Neurology, Changwon Fatima Hospital, Changwon, Korea; 4 Department of Neurology, Gangnam Severance Hospital, Severance Institute for Vascular and Metabolic Research, Yonsei University College of Medicine, Seoul, Korea; 5 Department of Neurology, Busan Paik Hospital, Inje University College of Medicine, Busan, Korea; 6 Department of Neurology, Sanggye Paik Hospital, Inje University College of Medicine, Seoul, Korea; 7 Department of Neurology, Kyung Hee University School of Medicine, Kyung Hee University Hospital at Gangdong, Seoul, Korea; 8 Department of Neurology, Ulsan University Hospital, Ulsan University College of Medicine, Ulsan, Korea; 9 Department of Neurology, Brain Research Institute, Keimyung University School of Medicine, Daegu, Korea; 10 Department of Neurology, Yonsei University College of Medicine, Seoul, Korea; University of Palermo, ITALY

## Abstract

**Introduction:**

Prior use of direct oral anticoagulants has been associated with reduced stroke severity in patients with non-valvular atrial fibrillation (NVAF). The aim of this study was to investigate the impact of prothrombin time (PT) and activated partial thromboplastin time (aPTT) on stroke severity in patients who were receiving dabigatran or rivaroxaban at the time of stroke onset.

**Materials and methods:**

We enrolled 107 patients with NVAF who developed acute ischemic stroke while on dabigatran or rivaroxaban and presented within 24 hours to nine hospitals between January 2014 and December 2018. The results of PT and aPTT assays were obtained within 24 hours of stroke onset in all patients. We analyzed PT and aPTT in relation to stroke severity and ischemic lesion volume using correlation and multivariable regression analyses.

**Results:**

Of the 107 patients included, 46 (43.0%) were on dabigatran and 61 (57.0%) were on rivaroxaban. In patients with prior dabigatran use, while aPTT was inversely correlated with admission National Institutes of Health Stroke Scale (NIHSS) score (r = -0.369, p = 0.012) and ischemic lesion volume (r = -0.480, p = 0.005), there was no correlation between PT and either of these variables. Multivariable analysis confirmed the existence of a significant independent inverse relationship between aPTT and NIHSS score at admission (B, -0.201; 95% confidence interval [CI], -0.370 to -0.032; p = 0.005) and between aPTT and ischemic lesion volume (B, -0.076; 95% CI, -0.130 to -0.023; p = 0.007). In patients with prior rivaroxaban use, neither PT nor aPTT was associated with admission NIHSS score or ischemic lesion volume in the correlation and multivariable analyses.

**Conclusions:**

In patients with NVAF who were receiving dabigatran, prolonged aPTT was associated with reduced stroke severity.

## Introduction

Non-valvular atrial fibrillation (NVAF) is the most common type of cardiac arrhythmia associated with an increased risk of cardioembolism [1]. Patients with cardioembolic stroke are known to have more severe neurological deficits and a poorer functional outcome than those with other types of ischemic stroke [2, 3]. Oral anticoagulants are used for primary and secondary stroke prevention in patients with NVAF [4–6]. In addition, prior use of oral anticoagulants has been shown to reduce stroke severity and subsequently improve functional outcome [7, 8].

In patients with NVAF who suffer an ischemic stroke, stroke outcome has been shown to differ according to the activity of oral anticoagulants [9]. Notably, specific coagulation tests, including diluted thrombin time and chromogenic anti-Xa assays, have demonstrated a strong correlation with plasma concentrations of direct oral anticoagulants (DOACs) [10]. However, the availability of these specialized tests is limited and they require personnel with laboratory experience, which restricts their implementation in clinical practice. Several experimental studies have shown that routine coagulation tests such as prothrombin time (PT) and activated partial thromboplastin time (aPTT) assays could provide qualitative estimates of the anticoagulant activity of dabigatran and rivaroxaban [10]. Therefore, stroke outcomes such as stroke severity and ischemic lesion volume could differ according to PT and aPTT in stroke patients on dabigatran or rivaroxaban. However, little is known about this issue, and further research is thus required.

The aim of this study was to investigate the impact of PT and aPTT on stroke severity in patients with NVAF who were receiving dabigatran or rivaroxaban at the time of stroke onset.

## Materials and methods

### Study population

We retrospectively reviewed the medical records of consecutive patients with NVAF who suffered an acute ischemic stroke while taking a DOAC and presented within 24 hours of symptom onset to the neurology department of nine hospitals between January 2014 and December 2018. After obtaining data on the type, dose, and time of last DOAC intake, we included 107 patients who were taking dabigatran or rivaroxaban. We excluded patients taking apixaban or edoxaban from this analysis because apixaban has been reported to be less sensitive to PT and aPTT assays than other DOACs [11–13] and edoxaban entered the Korean market in 2016, which was in the middle of our intended study period. In addition, patients were also excluded if they had taken their last DOAC dose more than 48 hours before the index stroke or if the time of last DOAC intake was unknown.

All patients underwent brain imaging, neurological examination, and routine coagulation tests, including PT and aPTT assays. The diagnosis of acute ischemic stroke was made by stroke neurologists and confirmed based on computed tomography or magnetic resonance imaging. The presence of AF was determined by electrocardiography. PT and aPTT assays were performed within 24 hours of symptom onset in all patients. These assays were carried out in the laboratory of each hospital according to the manufacturer’s instructions. In this study, PT was expressed in seconds (s) because its conversion to international normalized ratio (INR) does not correct for the variability resulting from the use of different thromboplastins [13, 14]. This study was approved by the institutional review board of each participating hospital and each board waived the need for patient consent (Pusan National University Hospital H-2004-035-090, Pusan National University Yangsan Hospital 05-2020-165, Severance Hospital 4-2019-1273, Gangnam Severance Hospital 3-2018-0046, Kyung Hee University Hospital KHNMC-2019-10-008, Changwon Fatima Hospital CHF-2019-12, Ulsan University Hospital 2020-08-006, Sanggye Paik Hospital 2019-04-018-001, and Busan Paik Hospital 2019-03-0024).

### Clinical data

We collected data on baseline characteristics and vascular risk factors such as hypertension, diabetes mellitus, dyslipidemia, and cigarette smoking. Patients were considered smokers if they had smoked within the 3-month period before admission. Data on each patient’s history of ischemic heart disease, ischemic stroke, peripheral arterial occlusive disease, and congestive heart failure were also obtained. Based on these data, we calculated the CHA_2_DS_2_-VASc score before the index stroke. Stroke subtype was determined according to the Trial of Org 10172 in Acute Stroke Treatment (TOAST) criteria [15].

Preadmission functional status was determined using the modified Rankin Scale (mRS), and initial stroke severity was assessed according to the National Institutes of Health Stroke Scale (NIHSS) score at admission.

We obtained the results of blood tests performed at admission, including lipid and creatinine levels. Serum creatinine clearance (CrCl) was calculated using the Cockcroft-Gault equation.

Patients were categorized into two groups according to the dose of DOAC and level of CrCl. The standard-dosed group included patients receiving dabigatran 150 mg twice daily (110 mg twice daily in patients with CrCl 30−49 mL/min) or rivaroxaban 20 mg once daily (15 mg once daily in patients with CrCl 15−49 mL/min). Therefore, patients who were receiving a DOAC dose lower than the abovementioned recommended dose were included in the under-dosed group. Data on the concomitant use of antiplatelet agents or statins with dabigatran or rivaroxaban were also obtained.

### Radiologic data

Ischemic lesion volume was measured based on initial diffusion-weighted imaging (DWI) using MIPAV software (version 8.0.2., http://mipav.cit.nih.gov). For this analysis, we excluded patients who underwent DWI after acute reperfusion therapy (n = 21), those whose DWI were unavailable (n = 5), those without ischemic lesions (n = 3), and those with artifacts (n = 3) or hemorrhagic transformation (n = 3) on DWI. Finally, 72 (67.3%) patients were included in the volumetric analysis. Two observers (H.-J.C. and Y.J.K.) manually outlined the hyperintense lesions on the initial DWI, and the acute ischemic lesion volume was automatically calculated by multiplying the lesion area in each section by the slice thickness. Acute ischemic lesion volume was measured independently by the two observers, and the mean value was then calculated.

### Statistical analysis

Categorical variables were expressed as frequency (percentage). Continuous variables were presented as median (interquartile range [IQR]) and were compared using the Mann-Whitney U test. We analyzed the correlation between PT/aPTT and admission NIHSS score and between PT/aPTT and ischemic lesion volume using Spearman's correlation test. To minimize bias owing to the NIHSS scores being restrained by lower and upper bounds, we conducted univariable and multivariable Tobit regression analyses to determine the factors independently associated with admission NIHSS score. Multivariable linear regression analysis was performed to identify the independent variables associated with ischemic lesion volume. Natural log transformation of the ischemic lesion volume was performed to reduce skewness and obtain a distribution more appropriate for correlation and multivariable linear regression analyses. The results were presented as B (95% confidence interval [CI]). P-values <0.05 were considered statistically significant. All statistical analyses were performed with SPSS for Windows (version 23.0, IBM Corp., Armonk, NY, USA) and R (version 3.1.0., http://www.R-project.org).

## Results

### Baseline characteristics

The median age of the 107 patients enrolled in this study was 75.0 years (IQR, 68.0−81.0), and 53 (49.5%) patients were male. The median time from symptom onset to hospital arrival and time from symptom onset to the acquisition of PT and aPTT results were 3.6 h (IQR, 1.2−11.2) and 5.4 h (IQR, 2.3−12.7), respectively. Dabigatran and rivaroxaban were prescribed in 39 (36.4%) patients for primary stroke prevention and in 68 (63.6%) patients for secondary stroke prevention. The median NIHSS score on admission was 7.0 (IQR, 3.0−15.0) and the median preadmission CHA_2_DS_2_-VASc score was 5.0 (IQR, 3.0−6.0). The median PT and aPTT were 13.4 s (IQR, 11.7−15.0) and 34.0 s (IQR, 29.9−40.6), respectively.

Of the 107 patients, 46 (43.0%) received dabigatran and 61 (57.0%) received rivaroxaban. Table 1 provides the baseline characteristics of patients in the dabigatran and rivaroxaban groups. The PT of the rivaroxaban group was significantly longer than that of the dabigatran group [14.2 s (IQR, 11.8−17.1) vs. 12.9 s (IQR, 11.6−14.2), p = 0.010]. On the contrary, the dabigatran group had a significantly prolonged aPTT compared with the rivaroxaban group [37.3 s (IQR, 29.2−46.7) vs. 33.3 s (IQR, 30.0−38.8), p = 0.034].

**Table 1:** Baseline characteristics of patients receiving dabigatran or rivaroxaban.

	Dabigatran (N = 46)	Rivaroxaban (N = 61)
Male	26 (56.5)	27 (44.3)
Age, years	74.5 (64.8–79.3)	75.0 (68.0–81.5)
Onset to arrival, hours	3.6 (0.7–12.5)	3.7 (1.3–11.1)
Onset to PT/aPTT results, hours	5.5 (1.9–13.7)	5.2 (2.6–11.9)
Preadmission mRS score	0.0 (0.0–1.0)	0.0 (0.0–2.0)
Stroke subtype		
Cardioembolic	41 (89.1)	50 (82.0)
Undetermined	5 (10.9)	11 (18.0)
Risk factors		
Hypertension	33 (71.7)	47 (77.0)
Diabetes	16 (34.8)	15 (24.6)
Dyslipidemia	14 (30.4)	17 (27.9)
Smoking	9 (19.6)	2 (3.3)
Previous ischemic heart disease	10 (21.7)	16 (26.2)
Previous ischemic stroke	30 (65.2)	38 (62.3)
CHA_2_DS_2_-VASc score	4.5 (3.0–6.0)	5.0 (4.0–6.0)
Under-dosed DOAC	19 (41.3)	16 (26.2)
Reperfusion therapy	7 (15.2)	14 (23.0)
Concomitant medication		
Antiplatelet agent	2 (4.3)	11 (18.0)
Statin	27 (58.7)	29 (47.5)
Laboratory findings		
PT, s	12.9 (11.6–14.2)	14.2 (11.8–17.1)
aPTT, s	37.3 (29.2–46.7)	33.3 (30.0–38.8)
Total cholesterol, mmol/L	3.7 (3.2–4.4)	3.7 (3.1–4.5)
LDL cholesterol, mmol/L	2.2 (1.7–2.7)	2.1 (1.5–2.9)
Creatinine clearance, mL/min	67.9 (50.8–82.9)	61.2 (41.4–75.8)

Values are number (column %) or median (interquartile range).

PT, prothrombin time; aPTT, activated partial thromboplastin time; mRS, modified Rankin Scale; DOAC, direct oral anticoagulant; LDL, low-density lipoprotein.

### Dabigatran group

**Fig 1:**
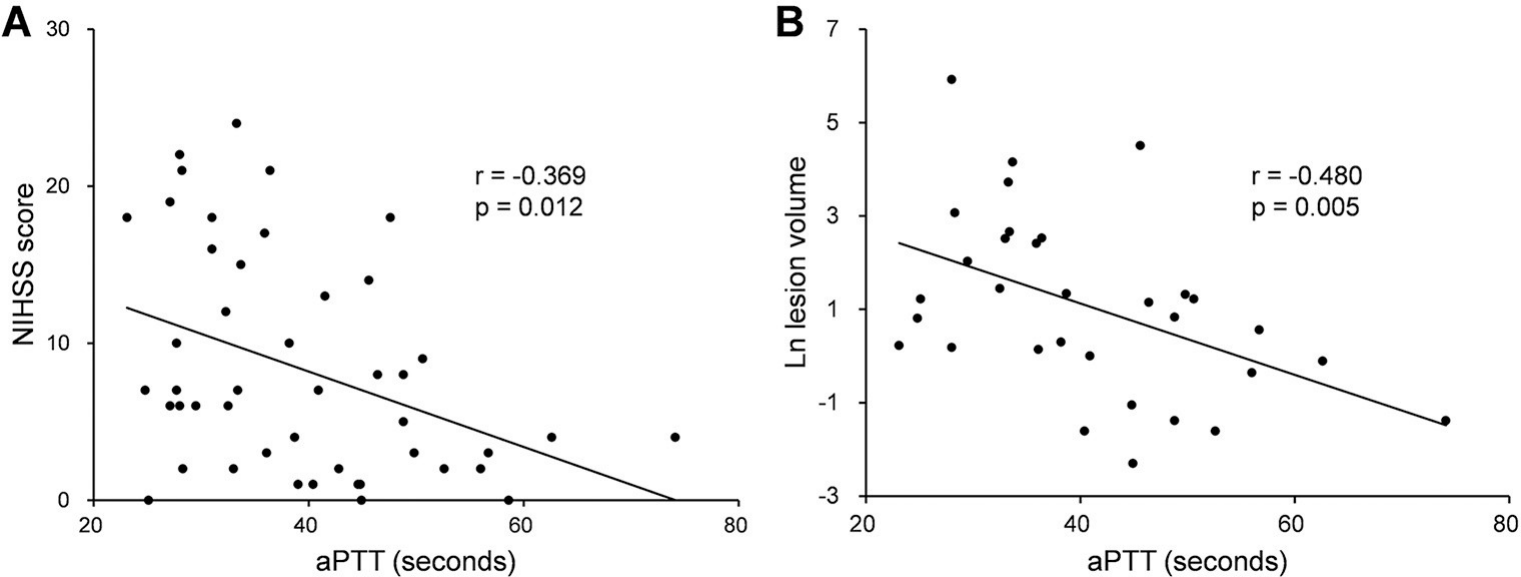
Correlations of aPTT in patients with prior dabigatran use. Scatter plots show the inverse associations between aPTT and admission NIHSS score (A) and between aPTT and acute ischemic lesion volume (B). aPTT, activated partial thromboplastin time; NIHSS, National Institutes of Health Stroke Scale.

The median NIHSS score was 6.5 (IQR, 2.0−14.3). The median ischemic lesion volume, which was measured in 32 (69.6%) patients, was 2.8 cm^3^ (IQR, 0.9−12.1). Spearman’s correlation analysis showed that there was a significant inverse correlation between aPTT and admission NIHSS score (r = -0.369, p = 0.012) and between aPTT and ischemic lesion volume (r = -0.480, p = 0.005; Fig 1 and S1 Table). However, PT did not correlate with admission NIHSS score or ischemic lesion volume (S1 Table). In the univariable Tobit regression analysis, hypertension (B, 4.924; 95% CI, 0.369 to 9.479; p = 0.034), previous ischemic heart disease (B, 5.962; 95% CI, 1.136 to 10.788; p = 0.015), and a shorter aPTT (B, -0.251; 95% CI, -0.425 to -0.077; p = 0.005) were associated with a higher NIHSS score at presentation. After adjusting for significant confounders, the multivariable Tobit regression analysis showed a statistically significant inverse association between aPTT and admission NIHSS score (B, -0.201; 95% CI, -0.370 to -0.032; p = 0.020; Table 2). A statistically significant inverse association between aPTT and ischemic lesion volume was also identified in the multivariable linear regression analysis (B, -0.076; 95% CI, -0.130 to -0.023; p = 0.007; Table 3).

**Table 2:** Multivariable Tobit regression analysis of the independent factors associated with stroke severity.

	Dabigatran		Rivaroxaban	
	B (95% CI)	P-value	B (95% CI)	P-value
Hypertension	3.509 (-0.695 to 7.713)	0.102	―	―
Previous ischemic heart disease	3.848 (-0.782 to 8.478)	0.103	―	―
Antiplatelet agent	―	―	7.050 (1.711 to 12.389)	0.010
PT	―	―	―	―
aPTT	-0.201 (-0.370 to -0.032)	0.020	―	―

B, standard coefficient; CI, confidence interval; PT, prothrombin time; aPTT, activated partial thromboplastin time.

**Table 3:** Multivariable linear regression analysis of the independent factors associated with ischemic lesion volume.

	Dabigatran		Rivaroxaban	
	B (95% CI)	P-value	B (95% CI)	P-value
Diabetes	―	―	0.981 (-0.223 to 2.186)	0.107
Antiplatelet agent	―	―	1.540 (0.125 to 2.956)	0.034
PT	―	―	―	―
aPTT	-0.076 (-0.130 to -0.023)	0.007	―	―

B, standard coefficient; CI, confidence interval; PT, prothrombin time; aPTT, activated partial thromboplastin time.

### Rivaroxaban group

The median NIHSS score was 9.0 (IQR, 3.0−16.0), and the median ischemic lesion volume, which was measured in 40 (65.6%) patients, was 6.9 cm^3^ (IQR, 2.4−19.9). In the Spearman’s correlation analysis, no significant correlations were identified between PT/aPTT and admission NIHSS score or ischemic lesion volume (S1 Table). The multivariable Tobit regression analysis showed that concomitant use of antiplatelet agents was significantly associated with a higher admission NIHSS score (B, 7.050; 95% CI, 1.711 to 12.389; p = 0.010; Table 2). Concomitant use of antiplatelet agents was also significantly associated with a greater ischemic lesion volume in the multivariable linear regression analysis (B, 1.540; 95% CI, 0.125 to 2.956; p = 0.034; Table 3). However, neither PT nor aPTT was associated with admission NIHSS score or ischemic lesion volume (Tables 2 and 3).

## Discussion

This study demonstrated an independent inverse relationship between aPTT on admission and stroke severity in patients with prior dabigatran use. However, PT was not associated with a reduction in stroke severity in patients on dabigatran or rivaroxaban.

In this study, the anticoagulant activity of dabigatran and rivaroxaban was assessed using PT and aPTT assays. Several experimental studies have focused on the application of coagulation tests for measuring DOAC activity [10]. Specific coagulation tests such as diluted thrombin time or ecarin-based assays could be used to accurately determine dabigatran activity as they strongly reflect the plasma concentration of this DOAC [16]. With respect to rivaroxaban, the chromogenic anti-Xa assay best reflects the plasma concentration of this DOAC [17]. However, these specific tests are not widely available in routine practice because they are typically performed in specialized coagulation laboratories and cannot be performed outside of daytime working hours. Indeed, in a previous study, less than half of all patients who presented with acute ischemic stroke while on DOAC therapy underwent specific coagulation tests in routine clinical practice [11]. Therefore, it would be helpful to understand the impact of DOACs on the results of routine coagulation tests utilized in clinical practice such as PT and aPTT assays.

Previous studies have shown a significant association between stroke outcomes and anticoagulant activity assessed using PT-INR in NVAF patients receiving warfarin [7, 8, 18]. In our study, a longer aPTT was significantly associated with reduced stroke severity and a smaller infarction volume in patients receiving dabigatran. aPTT has been experimentally reported to correlate with plasma dabigatran concentration in a curvilinear manner [19–22]. We speculate that a longer aPTT in patients on dabigatran may result in a smaller embolus size, enhanced embolus resolution, and reduced thrombus propagation, which may lead to decreased stroke severity [23, 24]. However, PT was not associated with stroke severity in patients on dabigatran, which correlates with previous experimental evidence showing that PT is less sensitive to dabigatran than aPTT [16, 20]. Previously, a prolonged aPTT (more than two times the upper limit of normal) was shown to be a predictor for bleeding events in patients on dabigatran [25]. However, there is a lack of data on the association between aPTT and ischemic stroke outcomes [26]. Our results may provide clinical evidence that the ability of dabigatran to reduce stroke severity is dependent on aPTT.

Previous experimental studies have revealed a linear correlation between PT and plasma rivaroxaban concentration [12, 17, 21]. However, aPTT may not be suitable for the evaluation of rivaroxaban activity because of its unresponsiveness and poor sensitivity to this DOAC [27]. Our study showed that neither PT nor aPTT was associated with a significant reduction in stroke severity in patients with prior rivaroxaban use. This discrepancy may be due to the fact that the thromboplastin reagents used in the PT assay were not identical among the participating institutes. In a previous study, different PT reagents showed highly variable sensitivities to rivaroxaban at the same plasma concentration [17]. Therefore, in our study, the ability of PT to accurately reflect the anticoagulant activity of rivaroxaban may have differed according to the reagents used.

There are limitations of this study that should be considered. First, this was a retrospective study with a small sample size. Therefore, unrecognized biases might have affected the validity of the results. The small number of patients included might be due to the low incidence of ischemic stroke in patients taking DOACs. Further larger prospective studies are needed to confirm the findings of our study. Second, we did not assess short-term or long-term outcomes due to the retrospective nature of this study. However, stroke severity on admission is an important predictor of functional outcomes in patients with ischemic stroke [28]. Although several studies have reported that anticoagulation therapy prior to ischemic stroke was associated with improved functional outcomes in patients with NVAF, this benefit was attenuated or no longer significant after adjusting for initial stroke severity [23, 29, 30]. Moreover, the long-term and even short-term outcomes of patients with ischemic stroke could be influenced by multiple factors after stroke onset. In this study, we sought to purely investigate the impact of the anticoagulant activity of two DOACs on stroke severity. Third, although several factors including inflammation and immune responses are known to be associated with stroke severity, these factors were not fully taken into consideration and adjusted for in this study [31, 32]. Finally, the interval between symptom onset and hospital arrival could have led to an underestimation of PT or aPTT at the time of stroke onset. However, we only included patients who presented within 24 hours of symptom onset. In particular, 33 patients (71.7%) in the dabigatran group and 47 (77.0%) in the rivaroxaban group presented to the hospital within 12 hours, which might have somewhat reduced the potential bias caused by the time lag between symptom onset and acquisition of the blood sample.

## Conclusions

Our study suggests that the ability of dabigatran to reduce stroke severity may be influenced by its anticoagulant activity in patients with NVAF. In addition, the aPTT assay can be easily used in clinical practice to estimate the anticoagulant activity of dabigatran. The accumulation of more clinical data is needed to support the findings of our study.

## Supporting information

S1 Table Correlations between PT/aPTT and stroke severity or ischemic lesion volume in patients on dabigatran or rivaroxaban. (DOCX)
