# Supplementary material for: Effects of dabigatran and rivaroxaban on stroke severity according to the results of routine coagulation tests
Source: PLoS One. 2020 Oct 12;15(10):e0240483. doi: 10.1371/journal.pone.0240483 (PMC7549802; doi:10.1371/journal.pone.0240483)
Supplement: S1 Table — (DOCX) [file pone.0240483.s001.docx]

**S1 Table. Correlations between PT/aPTT and stroke severity or ischemic lesion volume in patients on dabigatran or rivaroxaban**

|  | **PT** | | **aPTT** | |
| --- | --- | --- | --- | --- |
|  | **Coefficient** | **P-value** | **Coefficient** | **P-value** |
| Dabigatran | | | | |
| Admission NIHSS score | -0.152 | 0.313 | -0.369 | 0.012 |
| Ischemic lesion volume | -0.036 | 0.843 | -0.480 | 0.005 |
| Rivaroxaban | | | | |
| Admission NIHSS score | -0.062 | 0.638 | -0.188 | 0.147 |
| Ischemic lesion volume | -0.084 | 0.605 | -0.047 | 0.774 |

PT, prothrombin time; aPTT, activated partial thromboplastin time; NIHSS, National Institutes of Health Stroke Scale
